# Real-World Comparison of Cardiac MRI and Nuclear Perfusion Imaging for Myocardial Scar Detection

**DOI:** 10.1016/j.jacadv.2026.103303

**Published:** 2026-10-05

**Authors:** Ibolya Csecs, Ioannis Kyriakoulis, Ahmed Ibrahim Ahmed, Catherine X. Wright, Damianos Kokkinidis, Edward J. Miller, Albert J. Sinusas, Attila Feher

**Affiliations:** a Section of Cardiovascular Medicine, Department of Internal Medicine, Yale School of Medicine, New Haven, Connecticut, USA; b Faculty of Medicine, School of Health Sciences, University of Thessaly, Larissa, Greece; c Department of Radiology and Biomedical Imaging, Yale School of Medicine, New Haven, Connecticut, USA

**Keywords:** cardiac magnetic resonance imaging, late gadolinium enhancement, major adverse cardiac events, myocardial perfusion imaging, myocardial scar, positron emission tomography

## Abstract

**Background:**

Cardiovascular magnetic resonance late gadolinium enhancement (CMR-LGE) is the reference standard for scar detection, but data comparing LGE with fixed perfusion defects (FPD) on nuclear myocardial perfusion imaging (MPI) are limited.

****Objectives**:**

This study aimed to evaluate concordance between myocardial scar detection by CMR-LGE and nuclear MPI.

**Methods:**

In this retrospective single-center study, patients undergoing contrast CMR and nuclear MPI within 1 year were included: single-photon emission computed tomography (SPECT) with CT attenuation correction (SPECT^AC^, n = 328), SPECT without attenuation correction (SPECT^NoAC^, n = 246), or 82-rubidium-positron emission tomography (PET) (n = 240). Scar was assessed by CMR-LGE and FPD on MPI. Minimal resting myocardial blood flow (MBF^Min^) was determined for PET. Patients with myocardial infarction or revascularization between studies were excluded. Associations of LGE and FPD with major adverse cardiovascular events (MACE) were analyzed.

**Results:**

LGE was present in 354 patients (43%), predominantly nonischemic, whereas nuclear MPI detected scar less frequently (30%, *P* < 0.01). Nuclear scar was associated with ischemic LGE (OR: 5.86; 95% CI: 3.64-9.43; *P* < 0.001), but not nonischemic LGE. PET demonstrated higher specificity than SPECT for detecting ischemic and ≥50% transmural LGE (*P* < 0.01). Patients with ≥50% transmural LGE had lower MBF^Min^ (0.61 [0.53-0.68] vs 0.80 [0.74-0.85] mL/min/g; *P* < 0.001). Patients with both LGE and FPD had higher MACE risk than patients without these features (HR: 4.95 [95% CI: 3.01-8.11]; *P* < 0.001).

**Conclusions:**

MPI detected myocardial scar less frequently than CMR-LGE, with PET providing greater specificity than SPECT for ischemic and transmural scar. Combined LGE and FPD identified patients at highest risk of adverse outcomes, supporting PET MPI for myocardial scar characterization.

Myocardial perfusion imaging (MPI) is recommended by cardiovascular guidelines for the detection of obstructive coronary artery disease (CAD) by identifying reversible perfusion defect (RPD) representing myocardial ischemia and fixed perfusion defect (FPD) indicating myocardial scarring or prior myocardial infarction (MI).^1^ The extent and location of myocardial scarring vary significantly among patients, and its accurate assessment is crucial given the associated malignant arrhythmias and increased mortality risk.^2^, ^3^, ^4^ Different MPI techniques vary in their accuracy for detecting myocardial scar. Single-photon emission computed tomography (SPECT) MPI is the most widely used nuclear imaging method, but its performance could be severely impacted by attenuation artifacts. Attenuation correction (AC) by computed tomography has been shown to increase diagnostic accuracy including the detection of FPD, and it is the recommended technique over noncorrected imaging.^5^, ^6^, ^7^, ^8^ Positron emission tomography (PET) MPI is generally considered superior to SPECT MPI given its higher spatial resolution, shorter scan times, better image quality with reduced attenuation artifacts, and greater accuracy in detecting CAD. While robust literature supports the excellent diagnostic performance of PET to detect myocardial ischemia, there are limited data available on identifying myocardial scar burden apparent as an FPD on resting images.

Late gadolinium enhancement (LGE) on cardiovascular magnetic resonance (CMR) imaging is the gold standard method to detect myocardial replacement fibrosis or scar. Robust literature describes the association between the presence of LGE in the left ventricle (LV) and adverse events in patients with various cardiovascular conditions as well as healthy subjects with and without CAD.^9^, ^10^, ^11^ Higher scar burden correlates with worse outcomes.^12^ CMR imaging has the ability to detect replacement fibrosis with excellent accuracy in the entire myocardium: LGE in subendocardial to transmural location suggests ischemic origin, and LGE in subepicardial/midmyocardial location corresponds to nonischemic scar.

However, limited data are available on how the pattern of replacement fibrosis/scar detected as LGE correlates with the scar on MPI. Prior studies mainly report scar detection in patients with ischemic heart disease, and data on nonischemic scar detection with conventional MPI techniques remain unknown. Therefore, we aimed to investigate the diagnostic performance of 3 different clinical nuclear imaging techniques (SPECT MPI with or without AC and PET MPI) in the detection of myocardial fibrosis/scar pattern compared to CMR-LGE as the reference standard and to investigate their association with major adverse cardiovascular events (MACE) in a real-world tertiary care population.

## Methods

### Study participants

In this retrospective single-center study, we used the Yale Nuclear Database (n = 81,374 nuclear imaging studies) and selected patients who underwent both clinically indicated contrast CMR scan and nuclear MPI with either 99mTc-tetrofosmin SPECT or 82-rubidium (82Rb) PET within a 1-year period. Patients with MI or any revascularization between the 2 studies were excluded (Supplemental Figure 1). Baseline patient characteristics including age, sex, body mass index (BMI), race, comorbidities, and cardiovascular medications were recorded using the electronic medical records. The Institutional Review Board approved the study (2000026611). This study complied with the ethical standards of the responsible institution regarding human subjects and with the Helsinki Declaration.

### SPECT and PET image acquisition and analysis

Image acquisition of SPECT and 82Rb PET/CT was performed as previously described.^8^^,^^13^ Briefly, SPECT imaging was performed using 99mTc-tetrofosmin with one of the following cameras: GE 530, GE 570C, GE 850, GE Discovery GE Infinia, GE Myosight/CPC, GE Ventri, Philips CardioMD, or Siemens eCam. Patients underwent either symptom-limited exercise treadmill stress testing or a pharmacologic stress test. Patients were selected for exercise or pharmacologic stress using clinical parameters. Pharmacologic stress was performed by intravenous injection of a 0.4 mg bolus of regadenoson over 30 seconds. During SPECT, attenuation correction CT (SPECTAC) was performed based on the camera capability at the performing site. Dynamic rest-stress 82Rb PET/CT MPI was performed on a whole body 2-dimensional/3-dimensional PET camera equipped with a 64-slice CT scanner (Discovery, GE Healthcare).

Nuclear myocardial perfusion images were analyzed using Invia Corridor 4DM v2017 (Ann Arbor, MI) and graded as normal, abnormal, or equivocal perfusion. The presence of RPD representing myocardial ischemia and FPD representing scar was identified for each study subject. The presence or absence of an FPD was determined from the original clinical MPI reports generated during routine care by board-certified cardiologists and/or radiologists in nuclear cardiology. No dedicated research re-review, consensus interpretation, or adjudication was performed. FPD status was based on the final clinical report. Summed stress score (SSS) and summed rest score (SRS) were calculated routinely as the sum of the respective scores of all 17 segments, and derived summed difference score (SDS) as the difference between SSS and SRS. In patients who underwent 82Rb PET/CT, stress and rest myocardial blood flow (MBF) and myocardial flow reserve were also reported. Polar plots were generated using the American Heart Association 17-segment model, and the segment with the lowest MBF (MBF^Min^) was identified for patient-level analysis.

### CMR image acquisition and analysis

CMR scans were performed on a 1.5-T clinical scanner (Aera or Avanto, Siemens) using standard clinical protocols as previously published.^14^ Steady-state free precession cine imaging with retrospective electrocardiographic gating was acquired in the 2-chamber, 3-chamber, and 4-chamber views, and in contiguous short-axis slices of the LV. Delayed images for LGE evaluation were acquired 8 to 10 minutes after administration of 0.2 mmol/kg gadolinium contrast agent (gadoterate meglumine). After performing an inversion scout sequence, LGE imaging was obtained using inversion recovery gradient echo.

CMR images were analyzed using cvi42 (Circle Cardiovascular Imaging Inc, version 6.1.3). LGE (present or absent) was evaluated visually by board-certified cardiologists/radiologists. LGE was described as ischemic (subendocardial, transmural) and nonischemic (mid-myocardial, subepicardial) based on the pattern of distribution. To ensure a consistent comparison of MPI techniques for detecting replacement fibrosis, only patients with definite LGE were classified as LGE-positive; 4 patients whose LGE was limited to a subtle, nonspecific insertion zone-only pattern were therefore considered LGE-negative. In this study, FPD and RPD were compared to definite LGE as the gold standard to evaluate replacement fibrosis. Scars involving ≥50% of the myocardial wall thickness were categorized as ≥50% transmural LGE, irrespective of whether the enhancement pattern was ischemic or nonischemic. In addition, the maximum transmurality of ischemic LGE was further divided into 4 groups: transmural LGE <25%; LGE 25% to 50%; transmural LGE ≥50% but <75%; and transmural LGE ≥75%. The extent of LGE was first estimated using the semiquantitative method as summing the LV segments with LGE, respectively. Finally, scar burden was also quantified on short-axis LGE images using cvi42. Endocardial and epicardial contours were manually traced, and LGE extent was quantified using threshold-based methods consistent with current recommendations and common practice. For ischemic LGE, scar burden was quantified using the full width half maximum method, with hyperenhancement defined as signal intensity >50% of the maximal signal intensity within the scar.^15^ For nonischemic LGE, scar burden was quantified using the 5-SD threshold method, with remote myocardium serving as the reference region. Scar burden was expressed as the percentage of total left ventricular myocardial mass demonstrating enhancement (LGE%).^15^^,^^16^

### Statistical analysis

The Kolmogorov-Smirnov test was used to assess the normal distribution of the data. Continuous normally distributed variables were reported as mean ± SD, and continuous non-normally distributed variables as median with an IQR. Categorical and ordinal variables were reported as frequencies or percentages. Comparisons between groups were made using the Student’s *t*-test or the Mann-Whitney *U* test as appropriate. The one-way analysis of variance (ANOVA) or Kruskal-Wallis test was used when comparing more than 2 groups. Diagnostic performance was assessed using sensitivity, specificity, positive predictive value, negative predictive value, overall accuracy, and positive and negative likelihood ratios (LR+ and LR−), with LGE on CMR serving as the reference standard. Cohen’s kappa (κ) to quantify agreement between nuclear scar on MPI and LGE on CMR was calculated separately for each imaging modality. To compare sensitivity and specificity, the 2-proportion z-test or Fisher exact test was used. To account for baseline differences between imaging modality groups, a multivariable logistic regression analysis was performed to identify factors associated with correct scar exclusion (true-negative classification). Imaging modality (PET, SPECT^AC^, and SPECT^NoAC^) was entered as the primary variable, with PET used as the reference category. Covariates were selected a priori based on clinically relevant differences observed between groups and included age, sex, race, BMI, diabetes mellitus, chronic kidney disease, LV ejection fraction (LVEF), and ischemic LGE. We calculated the OR to assess the association between the presence of LGE and nuclear scar/ischemia on a per patient level. Unadjusted survival analysis was conducted using the Kaplan-Meier method, and statistical comparisons were made using the log-rank test. The primary clinical outcome was MACE, defined as a composite of all-cause death, MI, or hospitalization for heart failure. Clinical events were ascertained through electronic medical record review. Follow-up time was calculated from the date of the first index imaging study to the first MACE event or the last available clinical follow-up. For survival analysis, for patients undergoing PET MPI, patients were classified using a lower-quartile MBFMin threshold of 0.6 mL/min/g to define nuclear scar positivity (eg, nuclear scar defined as FPD and/or MBFMin <0.6 mL/min/g). Alluvial diagrams were constructed to visualize the relationship between LGE characteristics and the corresponding likelihood of FPD detection on nuclear imaging. Statistical analyses were performed using IBM SPSS statistics (v. 31.0.0.0) and STATA (version 19, Stata Corp) software package. Secondary and subgroup analyses were considered exploratory, and no formal adjustment for multiple comparisons was performed. There were no missing data for the primary imaging variables, including LGE presence, LGE type, and nuclear scar. No imputation was performed, and complete-case analysis was used for analyses involving covariates with missing values. Statistical significance was defined as *P* < 0.05 for all analyses.

## Results

### Patient characteristics

A total of 814 patients (mean age 65 ± 10 years, 55% male) who underwent both a clinically indicated CMR scan and nuclear MPI within 1 year were included in the study (Table 1). The average interval between the 2 studies was 97 ± 5 days, and over two-thirds of the cohort underwent both imaging modalities within 3 months. For 595 patients, nuclear MPI was the first imaging modality, followed by a CMR scan. We separated 3 groups according to the type of nuclear imaging: 1) patients who underwent SPECT without AC (SPECT^NoAC^); 2) patients who had SPECT with attenuation correction (SPECT^AC^); and 3) patients with 82Rb PET/CT (Table 1). Importantly, no differences were found in rates of prior cerebrovascular accident, MI, or coronary revascularization (Table 1).

**Table 1:** Baseline Characteristics

	SPECT^NoAC^ (n = 246)	SPECT^AC^ (n = 328)	PET (n = 240)	*P* Value
Age (y)	65 ± 10	62 ± 13	61 ± 14	0.007
Male, n (%)	135 (55)	189 (58)	114 (48)	0.053
BMI (kg/m^2^)	30 ± 7	30 ± 11	34 ± 9	0.001
White, n (%)	205 (83)	220 (67)	154 (64)	0.001
Black, n (%)	21 (9)	73 (22)	56 (23)	
Other, n (%)	20 (8)	35 (11)	30 (13)	
Hypertension, n (%)	154 (63)	182 (55)	147 (61)	0.178
Hyperlipidemia, n (%)	138 (56)	148 (45)	115 (48)	0.029
Diabetes mellitus, n (%)	38 (15)	50 (15)	68 (28)	0.001
CKD, n (%)	7 (3)	27 (8)	27 (11)	0.002
Obesity, n (%)	34 (14)	46 (14)	62 (26)	0.002
History of MI, n (%)	17 (7)	16 (5)	11 (5)	0.453
History of revascularization, n (%)	22 (9)	21 (6)	18 (7.5)	0.519
History of CVA, n (%)	7 (3)	16 (5)	12 (5)	0.403

BMI = body mass index; CKD = chronic kidney disease; CVA = cerebrovascular accident; MI = myocardial infarction; PET = positron emission tomography; SPECT^AC^ = single-photon emission computed tomography with attenuation correction; SPECT^NoAC^ = single-photon emission computed tomography without attenuation correction.

### CMR imaging findings

LGE was detected in 354 patients (43%), which was similarly distributed among the 3 nuclear imaging groups: 42% of patients undergoing SPECT^NoAC^, 43% of patients undergoing SPECT^AC^, and 46% of patients undergoing PET (Table 2). In addition, the total number of LGE-positive segments was similar in between MPI groups, and LGE% was also comparable in between groups (Table 2). Regarding scar characteristics, the presence of nonischemic LGE was more common than ischemic LGE in each group. Ischemic LGE was more frequent in patients undergoing PET MPI when compared to patients undergoing SPECT^NoAC^ or SPECT^AC^. No significant differences were observed between MPI groups in the rates of nonischemic LGE or the prevalence of ≥50% transmural LGE.

**Table 2:** Cardiovascular Magnetic Resonance and Nuclear Imaging Findings

	SPECT^NoAC^ (n = 246)	SPECT^AC^ (n = 328)	PET (n = 240)	*P* Value
Presence of LGE, n (%)	103 (42)	141 (43)	110 (46)	0.654
Nr of segments with LGE	0 [0-2]	0 [0-2]	0 [0-2]	0.287
LGE% of LV mass	7.2 [4.0-12.0]	6.0 [3.0-10.0]	7.5 [4.0-11.0]	0.629
LGE pattern				
Ischemic LGE, n (%)	26 (11)	25 (7.6)	36 (15)	0.023
Nonischemic LGE, n (%)	81 (33)	116 (35)	80 (33)	0.562
≥50% transmural LGE, n (%)	36 (15)	33 (10)	37 (15)	0.115
SPECT/PET parameters				
Fixed perfusion defect, n (%)	79 (32)	103 (31)	61 (25)	0.198
Reversible perfusion defect, n (%)	28 (11)	79 (24)	43 (25)	0.001
SRS	4.0 [0-9.25]	2.0 [0-9.0]	4.5 [1.0-9.0]	0.089
SSS	2.0 [0-5.5]	2.0 [0-8.0]	3.0 [0.5-8.0]	0.698
SDS	0.5 [0-2.0]	1.0 [0-3.0]	0 [0-2.0]	0.116
Global MBF at stress (mL/min/g)	-	-	2.03 [1.60-2.57]	-
Global MBF at rest (mL/min/g)	-	-	0.90 [0.71-1.11]	-
MBF^Min^ at rest (mL/min/g)	-	-	0.74 [0.40-1.20]	-
Global MFR	-	-	2.20 [1.78-2.69]	-
LVEF (%)	53 ± 14	57 ± 14	58 ± 15	0.001

Continuous nonnormally districuted variables reported as median [IQR]. LVEF presented as mean ± SD.

LGE = late gadolinium enhancement; LV = left ventricle; LVEF = left ventricular ejection fraction; MBF = myocardial blood flow; MFR = myocardial flow reserve; SDS = summed difference score; SRS = summed rest score; SSS = summed stress score; other abbreviations as in Table 1.

### Nuclear imaging findings

Nuclear MPI detected myocardial scar less frequently in comparison to the presence of LGE regardless of the type of nuclear imaging technique: 32% of patients undergoing SPECT^NoAC^, 31% of patients undergoing SPECT^AC^, and 25% of patients undergoing PET were found to have FPD (Table 2). Less than 25% of the patients had myocardial ischemia, and the prevalence of RPD was the lowest in the SPECT^NoAC^ group. The semiquantitative summed scores were not different between groups, suggesting no significant difference in the extent of the scar. For patients undergoing PET, global MBF at rest and stress as well as global myocardial flow reserve are displayed in Table 2.

### Comparing patients with and without LGE

In the entire cohort, nuclear scar was more common in patients with LGE than in patients without LGE (41.2% vs 21.1%; OR: 2.63; 95% CI: 1.91-3.62; *P* < 0.001) (Figure 1). Nuclear scar was also associated with ischemic LGE and ≥50% transmural LGE (Figure 1). However, nuclear scar was not associated with nonischemic LGE (Figure 1).

**Figure 1:**
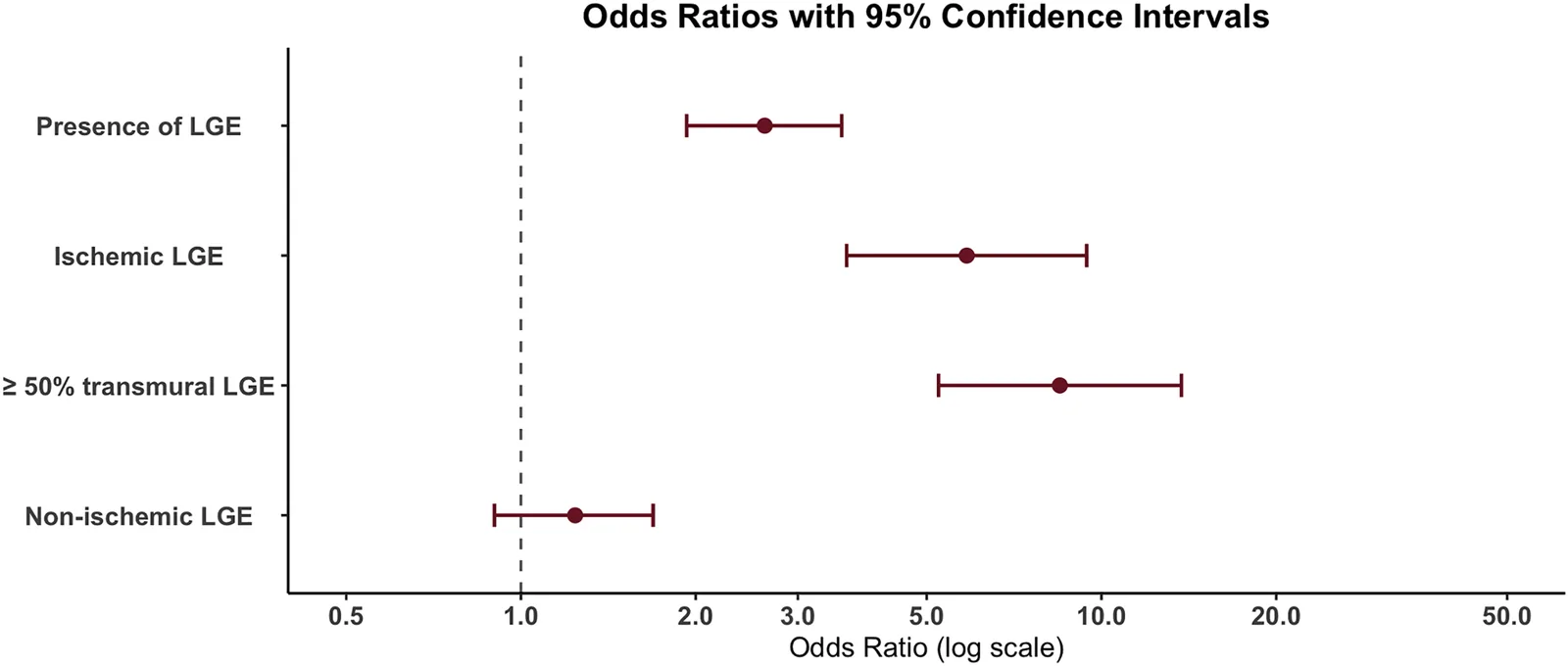
Association Between Late Gadolinium Enhancement and Nuclear Imaging Detected Scar Nuclear scar (red ORs) is associated with the presence of late gadolinium enhancement, ischemic late gadolinium enhancement, and ≥50% transmural late gadolinium enhancement. In contrast, nuclear scar is not associated with nonischemic late gadolinium enhancement. LGE = late gadolinium enhancement.

In the overall cohort, SRS did not differ significantly between patients with and without LGE. However, both SSS (6.3 [5.4-7.2] vs 4.0 [3.5-4.6] *P* = 0.002) and SDS (2.4 [2.0-2.9] vs 1.6 [1.4-2.0] *P* = 0.015) were higher among patients with LGE. Subgroup analysis demonstrated that these differences were entirely driven by the PET cohort, in which LGE-positive patients exhibited higher SSS and SDS compared with LGE-negative patients. In addition, LGE-positive patients in the PET cohort (n = 110) had lower global stress MBF (Figure 2). Patients with ≥50% transmural LGE (n = 37) had significantly lower MBF^Min^ compared to ≥50% transmural LGE negative cases (Figure 2). In PET cohorts, patients with greater LGE transmurality showed lower resting minimum MBF across the 4 LGE groups defined by increasing degrees of transmural scar (*P* < 0.001) (Supplemental Table 1).

**Figure 2:**
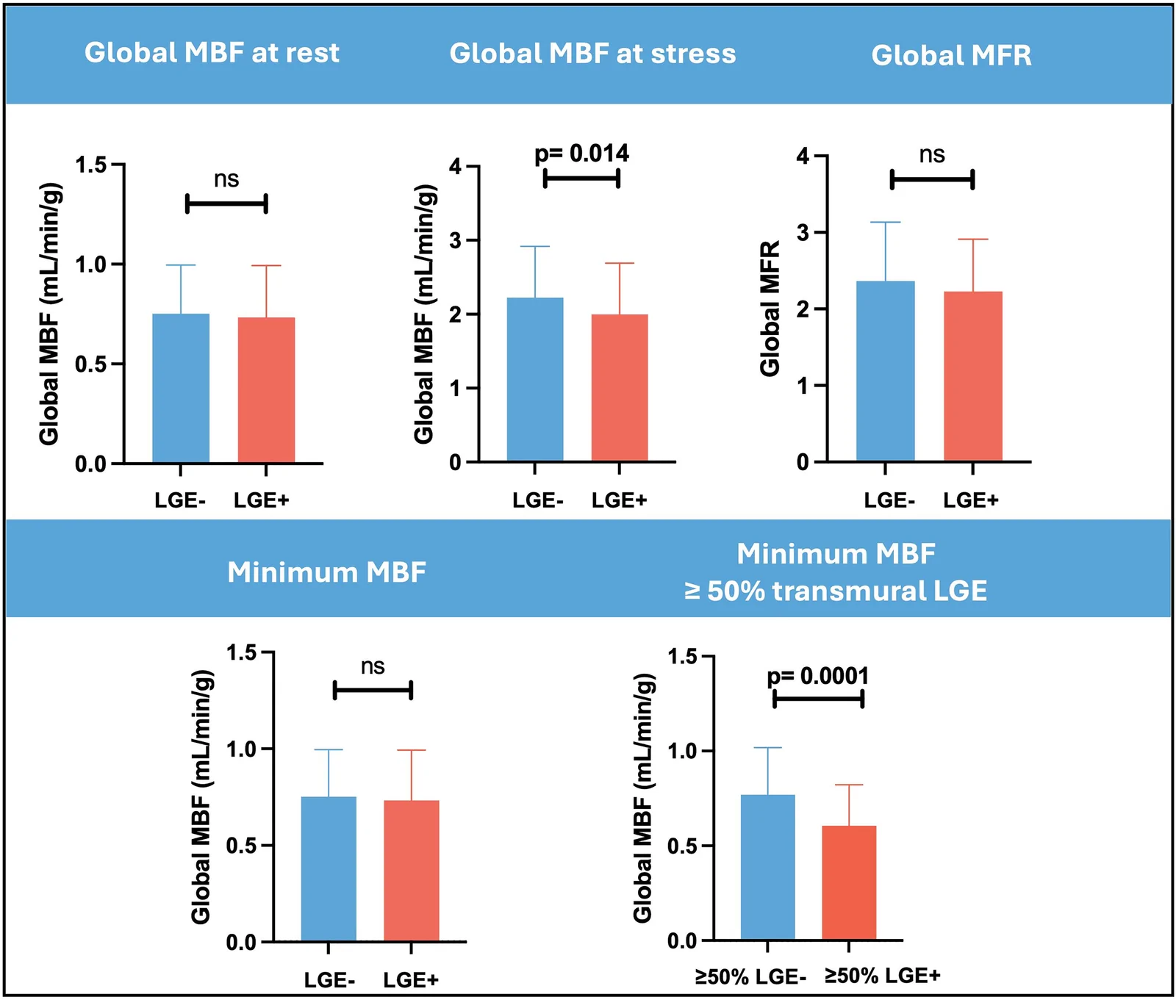
Differences in Myocardial Blood Flow in Patients With and Without Late Gadolinium Enhancement in Patients Undergoing Positron Emission Tomography/Computed Tomography Comparing patient with (n = 110) and without (n = 130) late gadolinium enhancement, the global resting myocardial blood flow was not different, but global myocardial blood flow at stress was lower in patients with late gadolinium enhancement. In addition, minimum myocardial blood flow was significantly lower in patients with ≥50% transmural late gadolinium enhancement. MBF = myocardial blood flow; MFR = myocardial flow reserve; other abbreviation as in Figure 1.

### Performance of different nuclear imaging in detecting scar and ischemia

Overall nuclear imaging had reasonable specificity however relatively low sensitivity to detect myocardial scar compared to LGE as a reference (Table 3). PET correctly identified true negative cases without any scar with an 84% specificity and identified true negatives without ischemic or ≥50% transmural LGE with ≥82% specificity. Comparing the performance of different nuclear modalities to each other, PET had significantly higher specificity to detect ischemic LGE and ≥50% transmural LGE compared to SPECT. Sensitivity was lower than specificity across all MPI techniques. Regarding nonischemic LGE, the sensitivity for detecting true-positive cases of nonischemic LGE was low across all nuclear imaging groups, whereas specificity was similar among MPI modalities (71%-72%). PET demonstrated higher overall diagnostic accuracy for detecting both ischemic LGE (80.4% vs 72.0% for SPECT^AC^ and 69.5% for SPECT^noAC^) and ≥50% transmural LGE (82.5% vs 73.2% and 71.8%, respectively), with additional diagnostic performance measures provided in Supplemental Table 2. Agreement between nuclear scar and any LGE was fair and comparable across modalities (κ = 0.206-0.215). PET demonstrated numerically greater agreement with ischemic LGE (κ = 0.403) and ≥50% transmural LGE (κ = 0.327) compared with both SPECT techniques, whereas agreement with nonischemic LGE was minimal across modalities (Table 4). Figure 3 displays a representative example of 2 patients. Patient 1 had subendocardial LGE which corresponds to an FPD on PET imaging consistent with ischemic scar. In contrast, patient 2 had both ischemic and nonischemic scar: the subendocardial LGE in the basal inferior and inferolateral segments identified as an FPD on PET images, although the midmyocardial LGE remained undetected on the nuclear images.

**Table 3:** Myocardial Scar Detection With Cardiovascular Magnetic Resonance and Nuclear Imaging

	SPECT^NoAC^ (n = 246)	SPECT^AC^ (n = 328)	PET (n = 240)	*P* Value PET vs SPECT^NoAC^	*P* Value PET vs SPECT^AC^
Nuclear scar vs LGE					
Sensitivity to detect LGE	44 [34-54]	43 [35-52]	36 [27-46]	0.275	0.269
Specificity to detect LGE	76 [68-83]	78 [71-83]	84 [76-90]	0.117	0.166
Nuclear scar vs ischemic LGE					
Sensitivity to detect ischemic LGE	58 [38-77]	72 [54-90]	69 [54-85]	0.334	0.829
Specificity to detect ischemic LGE	71 [65-77]	72 [67-77]	82 [77-87]	0.007	0.005
Nuclear scar vs nonischemic LGE					
Sensitivity to detect nonischemic LGE	39 [28-46]	37 [28-46]	20 [11-29]	0.011	0.012
Specificity to detect nonischemic LGE	71 [65-79]	72 [65-78]	72 [65-79]	0.927	0.834
Nuclear scar vs ≥50% transmural LGE					
Sensitivity to detect ≥50% transmural LGE	64 [47-78]	44 [28-59]	76 [60-87]	0.315	0.749
Specificity to detect ≥50% transmural LGE	73 [68-79]	71 [64-76]	84 [78-88]	0.001	0.001

Sensitivity and specificity displayed as % with 95% CI.

Abbreviations as in Tables 1 and 2.

**Table 4:** Calculated Cohen’s Kappa (κ) to Quantify Agreement Between Nuclear Scar and LGE Separately for Each Imaging Modality

LGE Phenotype	SPECT^NoAC^ κ	SPECT^AC^ κ	PET κ
Any LGE	0.206	0.215	0.209
Ischemic LGE	0.151	0.181	0.403
Nonischemic LGE	0.086	0.085	−0.078
≥50% transmural LGE	0.233	0.146	0.327

Abbreviations as in Tables 1 and 2.

**Figure 3:**
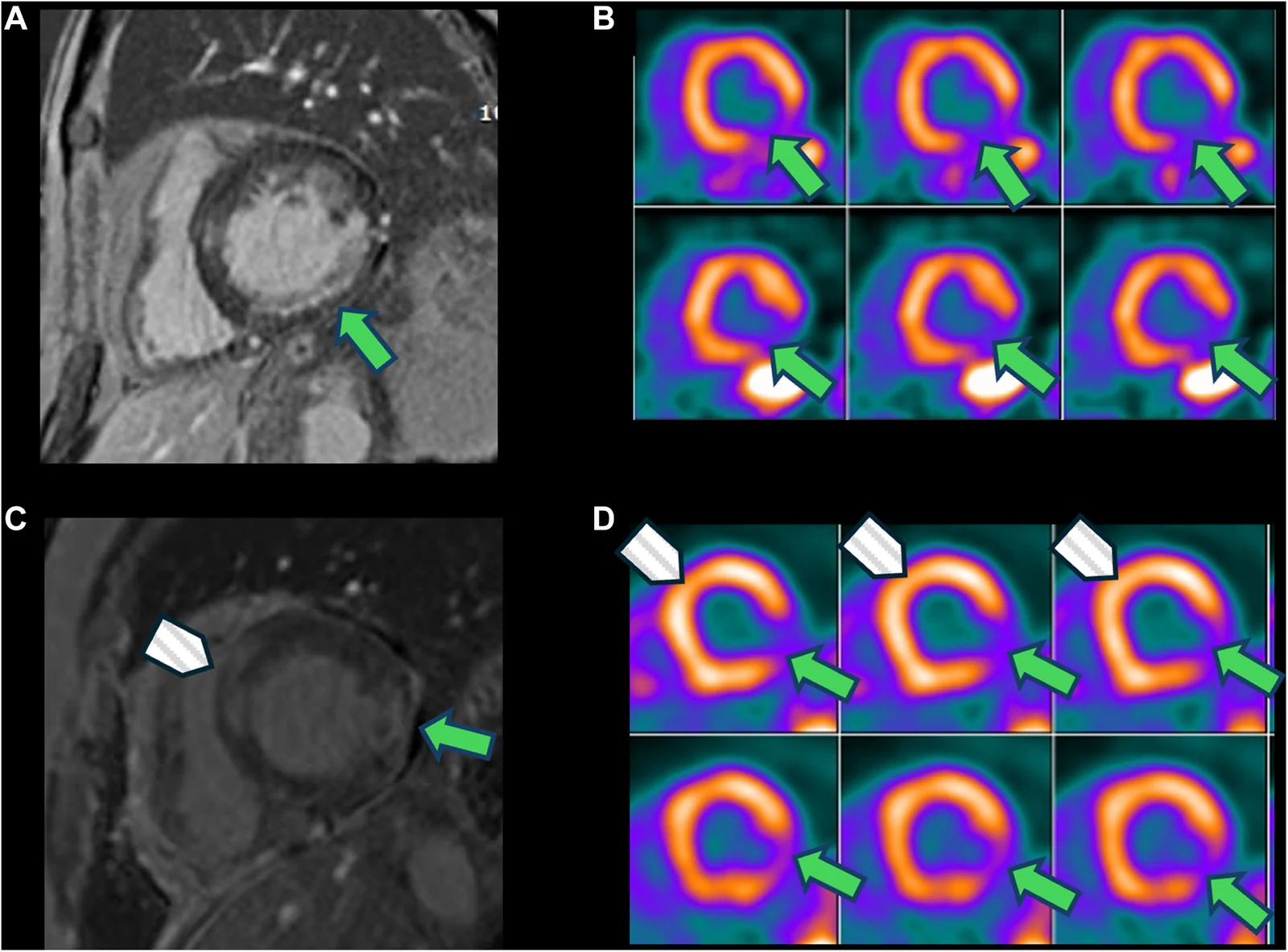
Representative Example of Patients Both Ischemic and Nonischemic Scar Patient 1 (A and B) demonstrates subendocardial late gadolinium enhancement in the basal inferior and inferolateral segments, which corresponds to a fixed perfusion defect on positron emission tomography imaging (green arrows), consistent with ischemic scar. In contrast, patient 2 (C and D) shows midmyocardial late gadolinium enhancement (C), indicative of nonischemic fibrosis, which remains undetected on positron emission tomography (D) (white arrows).

To account for baseline differences between modality groups, a multivariable logistic regression analysis was performed with true-negative classification as the dependent variable. After adjustment for age, sex, race, BMI, diabetes mellitus, chronic kidney disease, LVEF, and ischemic LGE, imaging modality remained independently associated with true-negative classification (Table 5). Compared with PET, both SPECT^AC^ (OR: 0.445; 95% CI: 0.297-0.666; *P* < 0.001) and SPECT^NoAC^ (OR: 0.449; 95% CI: 0.290-0.694; *P* < 0.001) demonstrated significantly lower odds of a true-negative result.

**Table 5:** Multivariable Logistic Regression for True-Negative Classification

	Adjusted OR [95% CI]	*P* Value
Age (per y)	0.981 [0.968-0.995]	0.007
Female	1.191 [0.856-1.655]	0.299
BMI	0.990 [0.977-1.003]	0.124
Diabetes mellitus	0.830 [0.542-1.270]	0.390
Chronic kidney disease	1.600 [0.854-2.997]	0.142
LVEF (%)	1.038 [1.026-1.051]	<0.001
Ischemic LGE	0.039 [0.012-0.129]	<0.001
Race	—	0.334
Black vs White	0.722 [0.466-1.120]	0.146
Other vs White	0.850 [0.499-1.448]	0.550
Imaging modality	—	<0.001
PET (reference)	1.00	—
SPECT-AC vs PET	0.445 [0.297-0.666]	<0.001
SPECT-noAC vs PET	0.449 [0.290-0.694]	<0.001

Abbreviations as in Tables 1 and 2.

A sensitivity analysis restricted to patients who underwent MPI and CMR within 90 days (n = 496 patients) yielded findings similar to the primary analysis. Imaging modality remained independently associated with true-negative classification (overall modality effect *P* = 0.009), with effect estimates comparable to those observed in the full cohort. Detailed results are provided in Supplemental Table 3.

### Scar characteristics and extent and the performance nuclear imaging

Assessment of maximal LGE transmurality demonstrated a stepwise association with the presence of FPD on nuclear imaging (Central Illustration). Among patients with <25% transmural LGE, FPDs were observed only in a small proportion. In the 25% to 49% LGE group, approximately half of the patients exhibited a corresponding FPD. In contrast, most patients with ≥50% but <75% transmural LGE showed a matched FPD. Notably, all patients with ≥75% transmural LGE demonstrated an FPD on CT-attenuation corrected nuclear imaging modalities (SPECT^AC^ and PET). Majority of patients with subendocardial defects demonstrated an FPD on nuclear perfusion imaging; however, most patients with non-subendocardial LGE showed no FPD (Supplemental Figure 2). Additionally, subendocardial LGE was more commonly associated with FPD on nuclear imaging, mostly independent of total scar burden as assessed by the summed number of LGE-positive segments (Supplemental Figure 3).

**Central Illustration:**
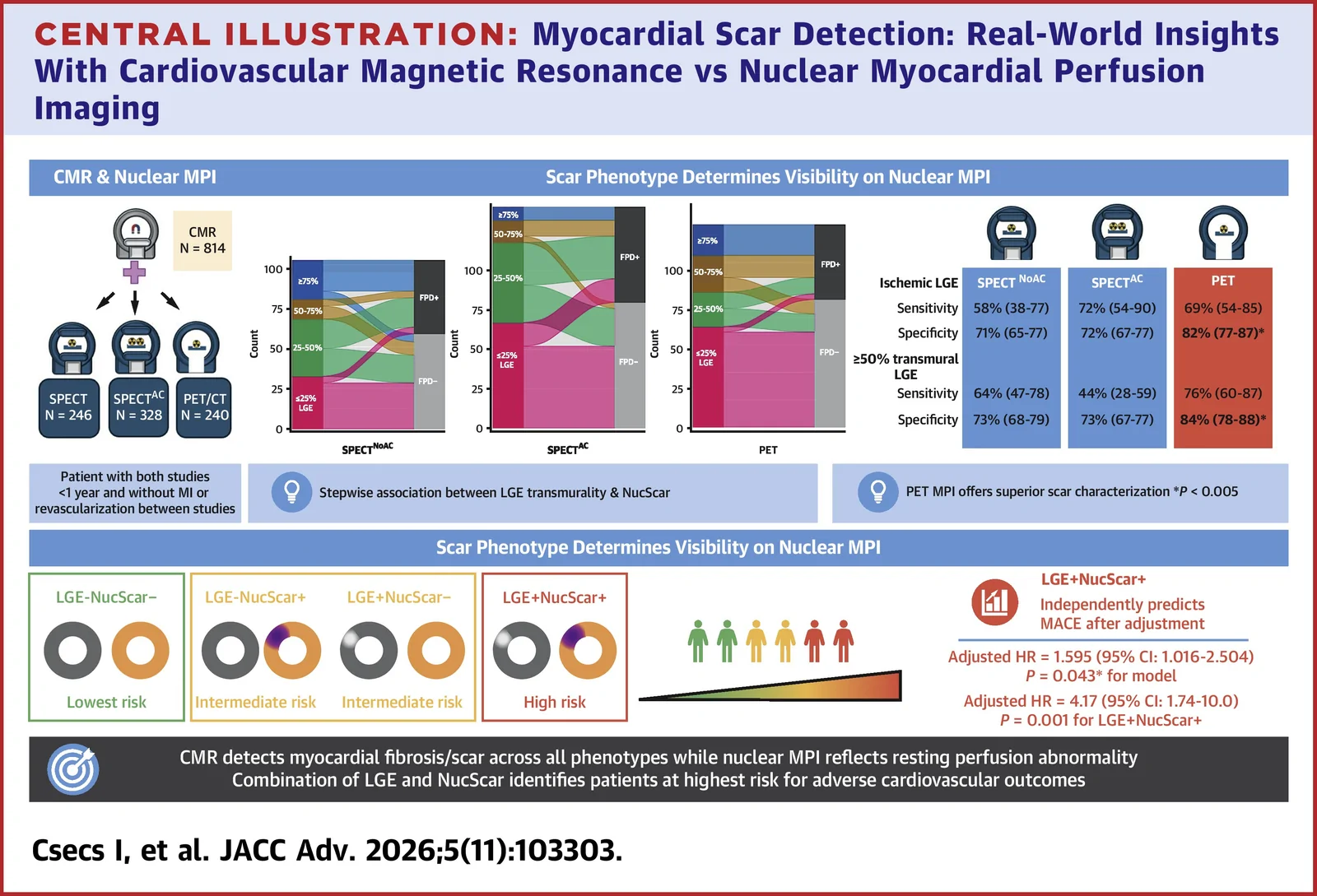
Myocardial Scar Detection: Real-World Insights With Cardiovascular Magnetic Resonance vs Nuclear Myocardial Perfusion Imaging Patients undergoing cardiovascular magnetic resonance and nuclear myocardial perfusion imaging within 1 year without intervening myocardial infarction or revascularization were evaluated for concordance of myocardial scar. Increasing late gadolinium enhancement transmurality was associated with greater detection of nuclear scar, while positron emission tomography demonstrated greater specificity for ischemic and ≥50% transmural late gadolinium enhancement compared with single-photon emission computed tomography. Cardiovascular magnetic resonance and nuclear myocardial perfusion imaging provided complementary prognostic information, with the combination of late gadolinium enhancement and nuclear scar identifying patients at highest risk for major adverse cardiovascular events. Overall, these findings demonstrate that scar phenotype influences its visibility on nuclear myocardial perfusion imaging and support the complementary roles of cardiovascular magnetic resonance–defined myocardial fibrosis and resting perfusion abnormalities in myocardial scar characterization and risk stratification. CMR = cardiovascular magnetic resonance; FPD = fixed perfusion defect; MACE = major adverse cardiovascular events; MPI = myocardial perfusion imaging; NucScar = nuclear scar; PET = positron emission tomography; SPECT-AC = attenuation-corrected single-photon emission computed tomography; SPECT-noAC = nonattenuation-corrected single-photon emission computed tomography; other abbreviation as in Figure 1.

The number of LGE-positive segments did not differ significantly between ischemic and nonischemic LGE groups, with a median of 3 segments (95% CI: 2-4) in the ischemic group and 2 segments (95% CI: 2-3) in the nonischemic group (*P* = 0.362). In contrast, LGE% was significantly greater in patients with ischemic LGE, with a median LGE% of 10% (95% CI: 8-12) compared with 6% (95% CI: 5-6.5) in patients with nonischemic LGE (*P* < 0.001).

To further evaluate the relationship between scar burden and MPI detection of nonischemic fibrosis, an additional analysis was performed using quantitative nonischemic LGE burden. High nonischemic LGE burden was defined as LGE ≥9% of left ventricular mass, corresponding to the upper quartile of the cohort. Patients with high nonischemic LGE burden were more likely to demonstrate an FPD than those with lower nonischemic LGE burden (32/44 [72.7%] vs 55/136 [40.4%], *P* = 0.043).

### Myocardial scar and clinical outcomes

During the median follow-up of 2.76 (1.8-4.2) years, a total of 114 patients (14%) experienced MACE. Patients who had both LGE and FPD had worse event-free survival when compared to those without any or only one of these features (*P* < 0.001) (Figure 4). Similar trend was observed when examining these trends per nuclear modality (Supplemental Figure 4). Univariate predictors of MACE are described in Table 6. In a multivariable Cox proportional hazards model adjusting for age, BMI, diabetes mellitus, chronic kidney disease, and LVEF, the combined imaging phenotype defined by the presence of LGE, FPD, and/or severely reduced resting MBF (LGE+ NucScar+) remained independently associated with MACE (HR: 1.595; 95% CI: 1.016-2.504; *P* = 0.043) (Table 7). In the conventional multivariable Cox models, nuclear scar remained independently associated with outcomes across all 3 models (HR: 1.66; 95% CI: 1.07-2.56; *P* = 0.023 with LGE presence; HR: 1.57; 95% CI: 1.00-2.46, *P* = 0.049 with ischemic LGE; and HR 1.57, 95% CI: 1.00-2.46, *P* = 0.049 with LGE segment burden), whereas the corresponding LGE measures were not independently significant. After accounting for identified nonproportional hazards with time-dependent terms, the association of nuclear scar was attenuated and was no longer statistically significant (Supplemental Table 4).

**Figure 4:**
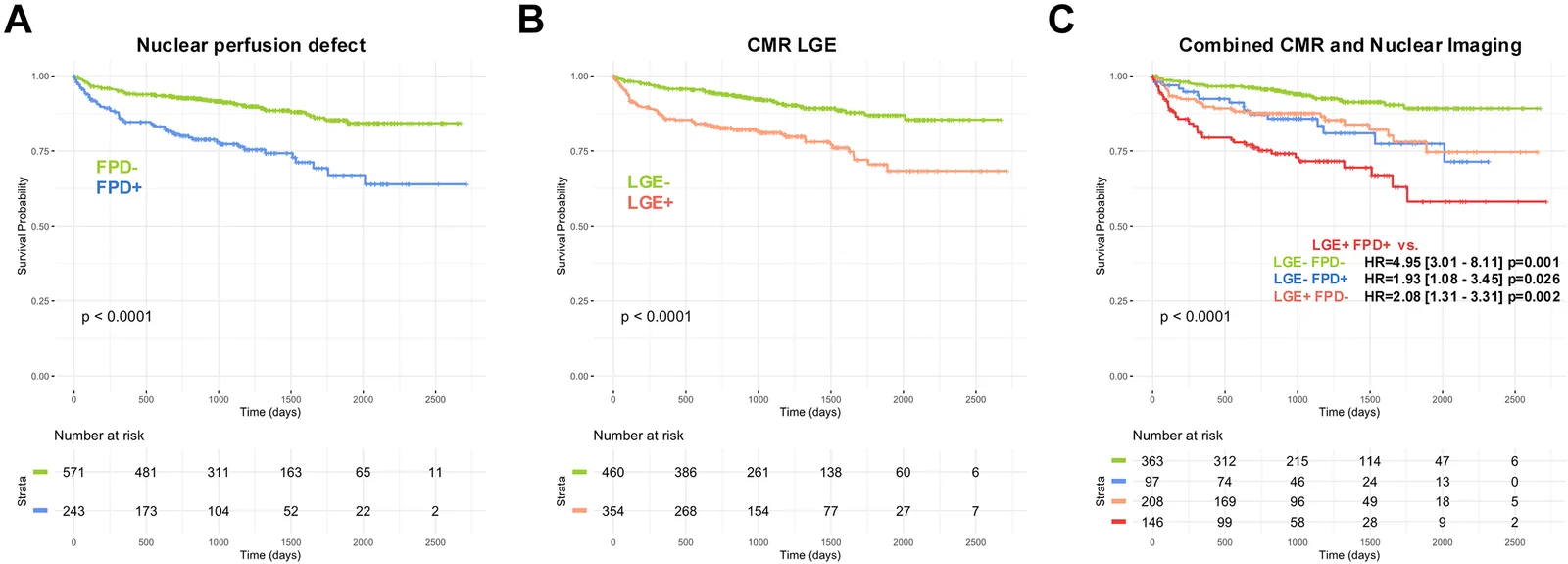
Survival Curves for Combined Imaging Phenotypes and Major Cardiovascular Outcomes Patients with concomitant late gadolinium enhancement and fixed perfusion defect demonstrated significantly worse survival and a higher incidence of the combined outcome of all-cause death, myocardial infarction, or heart failure hospitalization compared with patients without either feature (*P* < 0.001). FPD = fixed perfusion defect; other abbreviation as in Figure 1.

**Table 6:** Univariable Cox Proportional Hazards Analysis for MACE

	HR (95% CI)	*P* Value
Age	1.026 (1.009-1.043)	0.003
Female	0.860 (0.713-1.037)	0.115
BMI	1.000 (0.990-1.011)	0.980
CKD	3.195 (1.949-5.238)	<0.001
Diabetes mellitus	2.238 (1.519-3.295)	<0.001
Prior MI	3.157 (1.804-5.526)	<0.001
Prior revascularization	2.116 (1.229-3.643)	0.007
LVEF (%)	0.952 (0.942-0.963)	<0.001
LGE (%)	1.057 (1.037-1.078)	<0.001
Number of LGE-positive segments	1.152 (1.094-1.213)	<0.001
Any LGE	2.560 (1.748-3.749)	<0.001
Subendocardial LGE	2.652 (1.670-4.211)	<0.001
FPD	2.652 (1.836-3.831)	<0.001
Nuclear ischemia	1.932 (1.289-2.896)	0.001
SSS	1.061 (1.038-1.084)	<0.001
SRS	1.031 (1.005-1.058)	0.019
SDS	1.076 (1.032-1.122)	<0.001
Global MFR	0.514 (0.327-0.808)	0.004
LGE+ NucScar+ phenotype	3.533 (2.430-5.136)	<0.001

CAD = coronary artery disease; FPD = fixed perfusion defect; other abbreviations as in Tables 1 and 2.

**Table 7:** Multivariate Cox Proportional Hazards Analysis for MACE

	Adjusted HR (95% CI)	*P* Value
LGE+ NucScar+	1.595 (1.016-2.504)	0.043
Age	1.019 (1.002-1.037)	0.032
BMI	1.001 (0.988-1.014)	0.838
Diabetes mellitus	1.730 (1.159-2.583)	0.007
CKD	2.630 (1.572-4.397)	<0.001
LVEF	0.961 (0.948-0.974)	<0.001

NucScar = nuclear scar; other abbreviations as in Tables 1 and 2.

Additional Kaplan-Meier analysis demonstrated no significant difference in MACE-free survival between FPD− patients with and without nonischemic LGE (*P* = 0.307) (Supplemental Figure 5). In contrast, patients with FPD exhibited worse outcomes regardless of nonischemic LGE status, indicating that the prognostic signal was primarily driven by the presence of FPD.

## Discussion

Our study provides several observations investigating the performance of nuclear MPI in the detection of replacement fibrosis/scar compared to CMR-LGE as a reference in a large general cardiology referral cohort. In this study, nuclear imaging detected myocardial scar less frequently than CMR-LGE. Ischemic scar was more accurately detected by nuclear imaging, while nonischemic myocardial scarring remained “hidden” more often. When comparing different nuclear techniques, PET had higher specificity for ischemic and ≥50% transmural scar detection compared to SPECT with or without AC. Patients with ≥50% transmural scar had significantly lower MBF^Min^ with PET MPI. Finally, patients with both LGE and FPD more frequently experienced MACE.

### Comparisons with prior studies and detection of type of scars

Prior studies comparing scar detected on nuclear MPI to CMR-LGE in patients following MI found good correlation between ischemic, highly transmural LGE and FPD on MPI.^17^, ^18^, ^19^, ^20^ The hybrid PET/CMR study by Heiniger et al^21^ also included patients after acute myocarditis and investigated patients with ischemic (n = 50) and nonischemic cardiomyopathy (n = 50). In line with our observations, the authors of this study found that fibrosis/scar extent was underestimated with PET MPI, likely because of its low sensitivity to detect FPD in the subepicardial and mid-myocardial layers compared to subendocardial or transmural scars.^21^ Our results confirm these observations in a large cohort including 277 patients with nonischemic LGE. We also found that all 3 nuclear modalities (SPECT^NoAC^, SPECT^AC^, and PET) had a low ≤39% sensitivity to detect nonischemic scar. In addition, in this large general cardiology referral cohort, nonischemic LGE did not associate with FPD or RPD, while patients with ischemic LGE had approximately 6 times higher odds to have scar detected by nuclear MPI. As presented on the alluvial diagrams, both subendocardial involvement and increasing transmural extent of LGE were associated with greater visibility as FPDs, indicating a strong dependence of nuclear detectability on LGE depth and transmurality. These observations could be partially explained by the pathophysiological differences in transmural myocardial blood supply. Physiologically, the subendocardium, what MPI can evaluate most accurately, has a higher blood flow and is more vulnerable to ischemia compared to the outer subepicardial layers. Under resting condition, a relatively large FPD can remain masked if located in the mid-myocardium or subepicardium without connection to the inner subendocardial layer given the baseline higher blood flow in that location. In addition, nonischemic scars are more heterogeneous than ischemic, and even a near transmural nonischemic scar can be missed if the subendocardium remains unaffected. Transmural perfusion gradient, a novel quantitative measure calculated as a gradient of MBF from the endocardial to the epicardial layer, could be a promising tool to better understand the capability and main limitations of PET detecting not only ischemic but also nonischemic scar as well.^22^

### Performance of PET vs SPECT

In our study, LGE positive patients had a lower global MBF at stress, and the resting MBF^Min^ was measured at 0.58 (0.47-0.69) in areas of high transmural LGE, which is similar to previously published data.^23^ When comparing different nuclear MPI techniques, PET had higher specificity for ischemic and ≥50% transmural scar detection compared to SPECT. Because imaging modality was selected clinically rather than assigned randomly, differences in baseline characteristics represented a potential source of confounding. Reassuringly, the association between PET and improved specificity persisted after adjustment for demographic characteristics, cardiovascular risk factors, and ischemic LGE burden. This suggests that the higher specificity observed with PET may not be related to differences in baseline characteristics or ischemic scar burden; however, we cannot entirely exclude remaining confounders. All these findings underscore the importance of incorporating PET MPI over SPECT MPI into clinical practice for more precise myocardial scar characterization. This is particularly relevant for specific indications such as cardiac sarcoidosis, where accurate detection of scar with MPI is critical. Current consensus documents recommend combining PET or SPECT MPI with fluorodeoxyglucose metabolic imaging to identify perfusion–metabolism mismatch.^24^ The limited sensitivity of MPI, including PET MPI, for the detection of nonischemic LGE has important clinical implications. Nonischemic scar patterns, such as those encountered in cardiac sarcoidosis, frequently involve patchy mid-myocardial or subepicardial fibrosis that may not result in substantial resting perfusion abnormalities. As a result, the absence of a FPD does not exclude the presence of clinically significant myocardial fibrosis. These findings highlight the complementary roles of MPI and CMR for the detection and characterization of nonischemic scar.

### Prognostic implications

In this general cardiology referral cohort, the presence of myocardial scar tissue detected by either LGE or FPD was associated with an increased risk of death, MI or heart failure hospitalization. The combined LGE+/FPD+ phenotype conferred the highest risk, whereas patients negative for both markers had the most favorable outcomes. These results align with evidence from prior studies using SPECT or PET that FPD representing nonviable scar tissue is an independent predictor of MACE.^25^, ^26^, ^27^, ^28^ Furthermore, Kaplan-Meier analysis demonstrated significant differences in MACE-free survival among all 4 phenotypic groups. The substantial attenuation of the risk associated with the LGE+/FPD+ phenotype after multivariable adjustment suggests that conventional cardiovascular risk factors and LVEF account for a considerable proportion of the observed excess risk; however, the persistence of a significant association supports additional prognostic information from the combined imaging phenotype. Notably, patients with FPD+/LGE− and FPD−/LGE+ phenotypes exhibited distinct survival trajectories. These findings suggest that FPD provides prognostic information beyond the presence of myocardial scar alone. The incremental prognostic value of FPD likely reflects the fact that FPD and LGE provide complementary rather than redundant information regarding myocardial injury, remodeling, and disease severity. While LGE identifies structural replacement fibrosis, FPD may additionally reflect the functional consequences of myocardial injury, including chronic hypoperfusion, microvascular dysfunction, and adverse remodeling, thereby enhancing risk stratification. Furthermore, restricting the analysis to patients who underwent CMR and nuclear imaging within 90 days yielded findings consistent with the primary analysis, supporting the robustness of our results despite differences in the interval between imaging studies.

Interestingly, despite the limited sensitivity of MPI for detecting nonischemic LGE, the presence of nonischemic LGE alone was not associated with worse outcomes among patients without FPD. In contrast, the presence of FPD was consistently associated with poorer MACE-free survival. These findings suggest that resting perfusion abnormalities may identify a higher-risk myocardial phenotype and may explain why MPI retained prognostic utility despite its limited sensitivity for nonischemic scar detection. While larger disease-specific studies are needed, these results indicate that the prognostic implications of nonischemic LGE and perfusion abnormalities are not necessarily equivalent and may reflect distinct aspects of myocardial disease.

To our knowledge, this is the largest study comparing the predictive value of MPI scar in comparison to LGE. The additive prognostic value of combining structural (LGE-CMR) and functional (FPD-MPI) scar markers underscores the importance of multimodality imaging for risk stratification and integrating nuclear imaging and CMR in clinical decision-making, particularly for identifying patients at the highest risk who may benefit from aggressive management or advanced therapies.

### Study Limitations

This study has several limitations to be considered. First, the single center, nonrandomized, retrospective study design carries inherent limitations with the potential for selection bias. In this study, we report data on ^82^Rb PET/CT, and the results should be interpreted with caution when extrapolating to other tracers, including novel tracers with improved positron range such as ^18^F-flurpiridaz. The nuclear and CMR imaging studies were performed with a clinical indication and were not acquired simultaneously, introducing potential confounding related to temporal variation. Given the observational design, residual confounding from unmeasured factors cannot be completely excluded. Given the exploratory nature of the secondary and subgroup analyses and the absence of adjustment for multiple comparisons, these findings should be interpreted cautiously and warrant confirmation in future studies. Importantly, to minimize a potential change in scar pattern, we only included patients undergoing the 2 scans within a year interval and carefully excluded patients with MI or any revascularization in between the 2 studies. On the other hand, we would like to highlight that our study included a real-world population and is the largest study to date comparing scar pattern on nuclear MPI and CMR-LGE in a general cardiology referral cohort. Future prospective multicenter studies using hybrid PET/MR are needed to directly correlate perfusion, fibrosis, and metabolic activity for comprehensive scar characterization.

## Conclusions

In a large general cardiology referral cohort, nuclear MPI detected myocardial scar less frequently than CMR-LGE with higher specificity for ischemic and ≥50% transmural LGE detection in PET vs SPECT, emphasizing its clinical value for scar characterization. Patients with both LGE and FPD experienced a higher incidence of MACE, defined as the composite of all-cause mortality or MI or heart failure admission. These findings underscore the importance of incorporating PET MPI over SPECT into clinical practice for more precise myocardial scar characterization when available.

Perspectives **COMPETENCY IN MEDICAL KNOWLEDGE:** Myocardial scar burden is an important determinant of cardiovascular prognosis. PET provided greater specificity for ischemic and transmural myocardial scar than SPECT. LGE and FPDs provide complementary prognostic information.

**TRANSLATIONAL OUTLOOK:** Prospective studies should evaluate integrated PET-CMR approaches for scar assessment and risk stratification.

### Declaration of generative AI and AI-assisted technologies in the writing process

Artificial intelligence tools were used to assist with language editing and improving clarity; authors take full responsibility for the content.

## Funding support and author disclosures

The authors have reported that they have no relationships relevant to the contents of this paper to disclose.
