# Supplementary material for: Real-World Comparison of Cardiac MRI and Nuclear Perfusion Imaging for Myocardial Scar Detection
Source: JACC Adv. 2026 Oct 5;5(11):103303. doi: 10.1016/j.jacadv.2026.103303 (PMC13673032; doi:10.1016/j.jacadv.2026.103303)
Supplement: Supplementary file 1 [file mmc1.docx]

**Real-World Comparison of Cardiac MRI and Nuclear Perfusion Imaging for Myocardial**

**Scar Detection**

**Supplementary material**

**Supplementary Figure 1. Flow diagram showing cohort selection and all exclusion steps.**

**Supplementary Figure 2.**

**Relationship between late gadolinium enhancement (LGE) location and fixed perfusion defects on nuclear imaging.**

When LGE involved the subendocardium (Subendo LGE), the vast majority of cases demonstrated a corresponding fixed perfusion defect (FPD) on nuclear imaging. In contrast, LGE lesions without contact with the endocardial layer were largely undetectable on nuclear scans. Both subendocardial involvement and increasing transmural extent of LGE were associated with greater visibility as fixed perfusion defects, indicating a strong dependence of nuclear detectability on LGE depth and transmurality.

**Supplementary Figure 3.**

**Association between subendocardial late gadolinium enhancement (LGE) and fixed perfusion defects (FPD) on nuclear imaging**

Myocardial scars with subendocardial LGE involvement were more frequently associated with FPD on nuclear imaging. This association was observed largely independent of overall scar burden, as quantified by the summed number of LGE‑positive myocardial segments, indicating that scar transmurality rather than extent may be the more significant determinant of concordant nuclear imaging abnormalities.

**Supplementary Figure 4. Survival curves for combined imaging phenotypes and major cardiovascular outcomes (MACE) according to the three nuclear imaging techniques**

Survival analyses were performed separately for each nuclear imaging technique (SPECT, SPECT^AC^, and PET) using four combined imaging phenotypes based on the presence or absence of myocardial scar by nuclear imaging and late gadolinium enhancement (LGE). In SPECT cohorts, the four groups were defined using fixed perfusion defects (FPD) on nuclear imaging, in the PET cohort, nuclear scar positivity (NucScar) was defined using a lower‑quartile myocardial blood flow (MBF) threshold (< 0.6 mL/min/g) and/or the presence of FPD. Among LGE positive patients, 40 patients had FPD on PET, and an additional n=16 patients met the criteria having a MBF below the threshold resulting n=56 patients in LGE+NucScar+ group. Among LGE negative patients, 21 had FPD and an additional 36 patients met the criteria having a low MBF resulting n=57 patients in LGE-NucScar+ group. Significant differences in MACE‑free survival were observed among all four combined phenotypes across all three imaging modalities. Patients with evidence of scar on both LGE and nuclear imaging experienced the worst outcomes compared with the other groups.

**Supplementary Figure 5** Survival analysis for patients with non-ischemic late gadolinium enhancement. Patients without a fixed perfusion defect (FPD−), the presence of non-ischemic LGE was not associated with a significant difference in MACE-free survival (A). When patients were stratified by both FPD and non-ischemic LGE status, the presence of FPD was the dominant determinant of adverse outcomes, with both FPD-positive groups demonstrating worse survival than their FPD-negative counterparts (B).

**Supplementary Table 1.**

**Relationship between scar transmurality and myocardial blood flow.** In PET cohorts, patients with greater late gadolinium enhancement (LGE) transmurality showed lower resting minimum myocardial blood flow (MBF) across the four LGE groups defined by increasing degrees of transmural scar (p < 0.001)

|  | **N** | **MBF (mL/min/g)**  Median [95% CI] | **P value** |
| --- | --- | --- | --- |
|  |  |  |  |
| **Any LGE** | 110 | 0.73 [0.68 – 0.78] | < 0.0001 |
| **<25% LGE** | 54 | 0.81 [0.74 - 0.89] |  |
| **25-49% LGE** | 19 | 0.74 [0.64 – 0.86] |  |
| **50-75% LGE** | 17 | 0.62 [0.53 – 0.72] |  |
| **>/=75% LGE** | 20 | 0.58 [0.47 – 0.69] |  |

CI= confidence interval; LGE= late gadolinium enhancement; MBF= myocardial blood flow

**Supplementary Table 2.**

**Performance of different myocardial perfusion imaging (MPI) modalities in detecting fixed perfusion defect compared with late gadolinium enhancement (LGE)**

| **LGE reference** | **SPECT^NoAC^** | **SPECT^AC^** | **PET** |
| --- | --- | --- | --- |
| **Any LGE** |  |  |  |
| Sensitivity, % | 43.7 (34.5–53.3) | 43.3 (35.4–51.5) | 36.4 (28.0–45.7) |
| Specificity, % | 76.2 (68.6–82.5) | 77.5 (71.0–82.9) | 83.8 (76.6–89.2) |
| PPV, % | 57.0 (46.0–67.3) | 59.2 (49.6–68.2) | 65.6 (53.0–76.3) |
| NPV, % | 65.3 (57.8–72.1) | 64.4 (58.0–70.4) | 60.9 (53.6–67.7) |
| Accuracy, % | 62.6 (56.4–68.4) | 62.8 (57.5–67.9) | 62.1 (55.8–68.0) |
| LR+ | 1.84 (1.27–2.65) | 1.93 (1.39–2.67) | 2.25 (1.42–3.58) |
| LR− | 0.74 (0.61–0.90) | 0.73 (0.62–0.86) | 0.76 (0.65–0.89) |
| **Ischemic LGE** |  |  |  |
| Sensitivity, % | 57.7 (38.9–74.5) | 72.0 (52.4–85.7) | 69.4 (53.1–82.0) |
| Specificity, % | 70.9 (64.6–76.5) | 71.9 (66.6–76.7) | 82.4 (76.5–87.0) |
| PPV, % | 19.0 (11.9–29.0) | 17.5 (11.3–25.9) | 41.0 (29.5–53.5) |
| NPV, % | 93.4 (88.6–96.3) | 96.9 (93.7–98.5) | 93.9 (89.3–96.5) |
| Accuracy, % | 69.5 (63.5–74.9) | 72.0 (66.9–76.5) | 80.4 (74.9–84.9) |
| LR+ | 1.98 (1.34–2.92) | 2.57 (1.89–3.48) | 3.94 (2.73–5.68) |
| LR− | 0.60 (0.38–0.94) | 0.39 (0.21–0.73) | 0.37 (0.23–0.61) |
| **Non-ischemic LGE** |  |  |  |
| Sensitivity, % | 38.0 (28.1–49.0) | 36.8 (28.6–46.0) | 20.3 (12.7–30.8) |
| Specificity, % | 70.7 (63.4–77.0) | 71.5 (65.1–77.1) | 72.3 (65.0–78.5) |
| PPV, % | 38.0 (28.1–49.0) | 40.8 (31.8–50.4) | 24.6 (15.5–36.7) |
| NPV, % | 70.7 (63.4–77.0) | 68.0 (61.6–73.7) | 67.0 (59.9–73.5) |
| Accuracy, % | 60.2 (53.9–66.1) | 59.5 (54.1–64.6) | 56.2 (49.9–62.4) |
| LR+ | 1.29 (0.90–1.87) | 1.29 (0.94–1.78) | 0.73 (0.44–1.22) |
| LR− | 0.88 (0.72–1.07) | 0.88 (0.75–1.04) | 1.10 (0.95–1.28) |
| **≥50% transmural LGE** |  |  |  |
| Sensitivity, % | 63.9 (47.6–77.5) | 44.2 (28.1–59.0) | 75.7 (59.9–86.6) |
| Specificity, % | 73.3 (67.0–78.9) | 72.5 (67.2–77.2) | 83.7 (78.0–88.2) |
| PPV, % | 29.1 (20.3–39.9) | 18.4 (12.1–27.0) | 45.9 (34.0–58.3) |
| NPV, % | 92.2 (87.1–95.4) | 98.2 (95.5–99.3) | 95.0 (90.7–97.3) |
| Accuracy, % | 72.0 (66.0–77.2) | 73.2 (68.1–77.7) | 82.5 (77.2–86.8) |
| LR+ | 2.40 (1.72–3.34) | 3.00 (2.31–3.90) | 4.66 (3.24–6.68) |
| LR− | 0.49 (0.32–0.77) | 0.24 (0.10–0.59) | 0.29 (0.16–0.51) |

LGE= late gadolinium enhancement; LR= likelihood ratio; NPV= negative predictive value; PPV= positive predictive value; SPECT^AC^= single-photon emission computed tomography with attenuation correction; SPECT^NoAC^ = single-photon emission computed tomography without attenuation correction; PET= positron emission tomography. Sensitivity and specificity displayed as % with 95% confidence interval.

**Supplementary Table 3. Multivariable logistic regression analysis of true-negative classification in patients undergoing myocardial perfusion imaging (MPI) and cardiovascular magnetic resonance (CMR) studies within 90 days.** Results from the restricted cohort (n= 496) were similar to those observed in the full study population as imaging modality remained an independent predictor of true-negative classification

| **Variable** | **Adjusted OR [95% CI]** | **p-value** |
| --- | --- | --- |
| Age (per year) | 0.979 [0.963–0.995] | 0.009 |
| Female sex | 0.693 [0.466–1.031] | 0.070 |
| BMI | 0.983 [0.964–1.003] | 0.099 |
| Diabetes mellitus | 0.666 [0.390–1.139] | 0.138 |
| Chronic kidney disease | 1.471 [0.629–3.437] | 0.373 |
| Ischemic LGE* | — | 0.997 |
| Imaging modality |  | 0.009 |
| PET (reference) | 1.00 | — |
| SPECT-AC vs PET | 0.458 [0.278–0.754] | 0.002 |
| SPECT-noAC vs PET | 0.626 [0.364–1.075] | 0.089 |

**Supplementary Table 4/A**

| **Variable** | **Model 1: LGE presence HR (95% CI)** | **p** | **Model 2: Ischemic LGE HR (95% CI)** | **p** | **Model 3: LGE segments HR (95% CI)** | **p** |
| --- | --- | --- | --- | --- | --- | --- |
| Age | 1.017 (0.999–1.035) | 0.058 | 1.017 (1.000–1.035) | 0.052 | 1.017 (1.000–1.035) | 0.052 |
| BMI | 1.001 (0.988–1.014) | 0.861 | 1.003 (0.990–1.016) | 0.672 | 1.003 (0.990–1.016) | 0.672 |
| Sex | 0.796 (0.529–1.197) | 0.274 | 0.789 (0.524–1.188) | 0.256 | 0.789 (0.524–1.188) | 0.256 |
| Diabetes mellitus | **1.763 (1.181–2.634)** | **0.006** | **1.655 (1.104–2.481)** | **0.015** | **1.655 (1.104–2.481)** | **0.015** |
| CKD | **2.585 (1.540–4.338)** | **<0.001** | **2.584 (1.539–4.339)** | **<0.001** | **2.584 (1.539–4.339)** | **<0.001** |
| Prior revascularization | 1.505 (0.856–2.645) | 0.155 | 1.333 (0.742–2.398) | 0.336 | 1.333 (0.742–2.398) | 0.336 |
| LVEF | **0.963 (0.950–0.977)** | **<0.001** | **0.962 (0.949–0.976)** | **<0.001** | **0.962 (0.949–0.976)** | **<0.001** |
| Nuclear scar | **1.658 (1.074–2.559)** | **0.023** | **1.570 (1.002–2.459)** | **0.049** | **1.570 (1.002–2.459)** | **0.049** |
| LGE presence | 1.313 (0.843–2.044) | 0.228 | — | — | — | — |
| Ischemic LGE | — | — | 1.586 (0.941–2.673) | 0.084 | — | — |
| Number of LGE+ segments | — | — | — | — | 1.059 (0.993–1.129) | 0.081 |

**Supplementary Table 4/B**

| **Variable** | **Model 1: LGE presence** | **p** | **Model 2: Ischemic LGE** | **p** | **Model 3: LGE segments** | **p** |
| --- | --- | --- | --- | --- | --- | --- |
| Age | 1.310 | **<0.001** | 1.308 | **<0.001** | 1.315 | **<0.001** |
| BMI | 1.655 | **<0.001** | 1.644 | **<0.001** | 1.673 | **<0.001** |
| Sex | 0.854 | 0.498 | 0.838 | 0.447 | 0.849 | 0.481 |
| Diabetes mellitus | 1.722 | 0.024 | 1.689 | 0.029 | 1.672 | 0.029 |
| CKD | 1.842 | 0.035 | 1.804 | 0.043 | 1.786 | 0.047 |
| Prior revascularization | 1.069 | 0.833 | 1.091 | 0.783 | 1.071 | 0.830 |
| LVEF | 1.076 | **0.003** | 1.071 | **0.003** | 1.071 | **0.003** |
| Nuclear scar | 1.280 | 0.287 | 1.350 | 0.201 | 1.314 | 0.239 |
| LGE presence | 1.238 | 0.363 | — | — | — | — |
| Ischemic LGE | — | — | 1.545 | 0.076 | — | — |
| LGE segments | — | — | — | — | 1.044 | 0.241 |
| **Age × ln(time)** | **0.957** | **<0.001** | **0.946** | **<0.001** | **0.946** | **<0.001** |
| **BMI × ln(time)** | **0.920** | **<0.001** | **0.901** | **<0.001** | **0.900** | **<0.001** |
| **LVEF × ln(time)** | **0.983** | **0.005** | **0.973** | **<0.001** | **0.973** | **<0.001** |

BMI= body mass index; CAD= coronary artery disease; CKD= chronic kidney disease; FPD= fixed perfusion defect; HR= hazard ratio; LGE= late gadolinium enhancement; LVEF= left ventricular ejection fraction
